# Effect of Chlorogenic Acid Supplementation in MPTP-Intoxicated Mouse

**DOI:** 10.3389/fphar.2018.00757

**Published:** 2018-08-06

**Authors:** Saumitra S. Singh, Sachchida N. Rai, Hareram Birla, Walia Zahra, Gaurav Kumar, Mallikarjuna R. Gedda, Neeraj Tiwari, Ranjana Patnaik, Rakesh K. Singh, Surya P. Singh

**Affiliations:** ^1^Department of Biochemistry, Institute of Science, Banaras Hindu University, Varanasi, India; ^2^School of Biomedical Engineering, Indian Institute of Technology, Banaras Hindu University, Varanasi, India

**Keywords:** chlorogenic acid, Parkinson’s disease, oxidative stress, neuroinflammation, dopaminergic neuron, substantia nigra

## Abstract

Oxidative stress and neuroinflammation play a key role in dopaminergic (DA) neuronal degeneration, which results in the hindrance of normal ongoing biological processes in the case of Parkinson’s disease. As shown in several studies, on 1-methyl-4-phenyl-1,2,3,6-tetrahydropyridine (MPTP) administration, different behavioral parameters have suggested motor impairment and damage of antioxidant defence. Thus, some specific biological molecules found in medicinal plants can be used to inhibit the DA neuronal degeneration through their antioxidant and anti-inflammatory activities. With this objective, we studied chlorogenic acid (CGA), a naturally occurring polyphenolic compound, for its antioxidant and anti-inflammatory properties in MPTP-intoxicated mice. We observed significant reoccurrence of motor coordination and antioxidant defence on CGA supplementation, which has been in contrast with MPTP-injected mice. Moreover, in the case of CGA-treated mice, the enhanced expression of tyrosine hydroxylase (TH) within the nigrostriatal region has supported its beneficial effect. The activation of glial cells and oxidative stress levels were also estimated using inducible nitric oxide synthase (iNOS) and glial fibrillary acidic protein (GFAP) immunoreactivity within substantia nigra (SN) and striatum of MPTP-injected mice. Administration of CGA has prevented the neuroinflammation in SN by regulating the nuclear factor-κB expression in the MPTP-induced group. The significant release of certain pro-inflammatory mediators such as tumor necrosis factor-α and interleukin (IL)-1β has also been inhibited by CGA with the enhanced expression of anti-inflammatory cytokine IL-10. Moreover, reduced GFAP staining within the nigrostriatal region has supported the fact that CGA has significantly helped in the attenuation of astrocyte activation. Hence, our study has shown that CGA supplementation shows its therapeutic ability by reducing the oxidative stress and neuroinflammation in MPTP-intoxicated mice.

## Introduction

The second most commonly occurring neurodegenerative disorder, that is Parkinson’s disease (PD), is commonly found in elderly people of age 65 or older (Poewe et al., 2017). The number of patients with PD is increasing rapidly and is expected to double between 2005 and 2030 as the population grows older (Meireles and Massano, 2012). Degeneration of the dopamine-synthesizing neurons to an extent of about 50%–70% (Poewe, 2008) within substantia nigra pars compacta (SNpc) is the characteristic symptom of PD leading to motor impairments such as bradykinesia, postural instability, rigidity, and tremor at rest (Obeso, 2010). Although the exact pathobiology of the disease is not fully known, certain studies have shown that the major contributing factors in the PD progression are reactive oxygen species (ROS) and reactive nitrogen species (RNS) production, mitochondrial damage, inflammation, ubiquitin–proteasome system impairment, and accumulation of abnormally folded proteins (α-synuclein) (Gandhi and Wood, 2005). Consequently, pathways and cascading involved in the oxidative stress and inflammatory processes can be further investigated to be targeted therapeutically to stop the PD progression (Schapira et al., 2014). With regard to the role of inflammation in PD, it might worsen with age due to genetic aberrations, and some sporadic factors causing immune alteration lead to glial activation resulting from the neuronal injury. Several studies have proposed the role of inflammation in the progression of dopaminergic (DA) neuronal loss (Kanaan et al., 2010). The neuronal loss is promoted when the central inflammatory response is induced with the active peripheral inflammation in PD (Liu and Bing, 2011).

Astrogliosis and microgliosis are the two important characteristics involved in the pathobiogenesis of PD, resulting from the nonspecific neurodegeneration in the SNpc of patients with PD (Hirsch et al., 2003). Gliosis can be induced by different environmental and biological toxins such as lipopolysaccharides, rotenone, or MPTP, further leading to mitochondrial degeneration, death of DA neurons, and nuclear fragmentation in cellular and animal models (Langston et al., 1999; Herrera et al., 2000; Samantaray et al., 2007; Niranjan et al., 2010; de Oliveira et al., 2011). Glial cell activation contributes to the PD pathophysiology by releasing pro-inflammatory cytokines and neurotoxic factors, such as interleukin (IL)-1β, tumor necrosis factor-α (TNF-α), and RNS (e.g., nitric oxide [NO]), leading to the PD progression and triggering neurodegeneration (Block et al., 2007; Rappold and Tieu, 2010). However, some anti-inflammatory cytokines such as IL-10 help in rescuing the cells from damage during inflammation. IL-10 functions by inhibiting the inflammation due to its ability to decrease the inflammatory cytokine production. Moreover, different studies have shown the beneficial effects of IL-10 in various neuro-inflammatory diseases such as PD, stroke, and traumatic or excitotoxic spinal cord injury (Bethea et al., 1999; Brewer et al., 1999; Cua et al., 2001; Frenkel et al., 2005; Qian et al., 2006). Mitogen-activated protein kinase (MAPK) family, classified in major components: p38 MAPK, extracellular signal-regulated kinases (Erks), c-Jun N-terminal kinase (JNK) and nuclear factor-κB (NF-κB), are considered as the important signaling pathways responsible for activation of glial cells mediated through the release of pro-inflammatory cytokines (Lan et al., 2011; Qian et al., 2015). Therefore, blocking NF-κB and MAPK signaling pathways inhibit inflammatory processes, which can be used as therapeutic targets for preventing the neuronal damage in PD.

Various models of PD such as rotenone, 6-hydroxydopamine (6-OHDA), and 1-methyl-4-phenyl-1,2,3,6-tetrahydropyridine (MPTP) have shown an increased number of activated microglia in SN of brain (Kitamura et al., 1994; Kurkowska-Jastrzebska et al., 1999). MPTP has been used to induce acute model of sporadic PD in mice and non-human primates and acts as a neurotoxin that selectively destroys DA neurons in SN, causing severe PD symptoms in humans (Langston et al., 1983).

**FIGURE 1:**
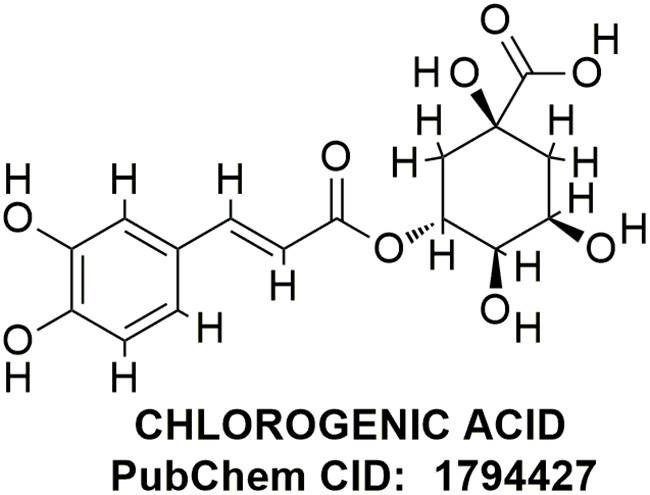
Chemical structure of chlorogenic acid.

Chlorogenic acid (CGA), a commonly occurring polyphenolic compound in various plants, is found particularly in green coffee beans containing about 5–12% of CGA by weight (Farah and Donangelo, 2006; Farah et al., 2008). CGA is an ester of *trans*-cinnamic acids (including caffeic acid, ferulic acid, p-coumaric acid) and quinic acid (**Figure 1**). It is commonly consumed by people and is found in different beverages and food items. It is mainly found in fruits such as apples, apricots, cherries, plums, and tomatoes and in vegetables such as potatoes. Wine, coffee, and tea are the most common beverages rich in CGA (Clifford, 1999). As CGA is found in a variety of foods and liquid refreshments, many researchers have attempted to investigate its nutritional benefits and physiological effects. The ability of CGA to reduce oxidative stress has been revealed in several studies. They all have shown that it can be used to induce antitumor activity and cardioprotective effects and may have neuroprotective effects (Han et al., 2010; Kwon et al., 2010; Cropley et al., 2012). Evidences suggest that CGA has multiple biological effects comprising antioxidant (Feng et al., 2005), neuroprotective (Huang et al., 2008; Kwon et al., 2010), and neurotrophic activities (Ito et al., 2008). Anti-inflammatory activity of CGA has been studied in different disease models, such as Lipopolysaccharide (LPS)-inflamed murine macrophage cells, mouse retinal inflammation model, carbon tetrachloride (CCl_4_)-induced liver fibrosis model, and LPS-inflamed keratinocytes (Lee S.A. et al., 2012; Shi et al., 2013; Hwang et al., 2014). A study has reported that endotoxin-induced inflammation has been significantly reduced by downregulating NF-κB pathway in raw 264.7 macrophages and mouse retinal inflammation model, which is responsible for the activation of genes resulting in the production of pro-inflammatory cytokines and adhesion molecules (Hwang et al., 2015). As a result, CGA has been much considered, and the demand of natural products or preparations containing CGA has been increased. Regardless of various studies suggesting the antioxidative and neuroprotective properties of CGA, no evidence of its antioxidative and anti-inflammatory effects in MPTP-injected mice has been shown. Therefore, we investigated the antioxidative and anti-inflammatory effects of CGA in MPTP-intoxicated mice. The results of our study have shown that CGA has significantly reduced the effect of MPTP intoxication by attenuating behavioral damage and increasing the expression of TH in DA neurons within the nigrostriatal region via its antioxidative and anti-inflammatory action. Moreover, it has also suppressed the production of cytokines such as TNF-α by inhibiting the NF-κB pathway.

## Materials and Methods

### Reagents and Antibodies

Chlorogenic acid, MPTP, and normal goat serum (NGS) were bought from Sigma–Aldrich (St. Louis, MO, United States). Acetic acid, ammonium chloride, bovine serum albumin (BSA), potassium chloride, disodium hydrogen phosphate, reduced nicotinamide adenine dinucleotide phosphate (NADPH), and sodium dihydrogen phosphate were procured from Sisco Research Laboratories (SRL, Mumbai, India). Protein estimation kit by Bradford GeNeiTM, hydrogen peroxide (H_2_O_2_), and potassium dichromate were purchased from Merck (Darmstadt, Germany), and sodium dodecyl sulphate (SDS), thiobarbituric acid (TBA), DABCO, and Griess reagent were obtained from HiMedia (Mumbai, India). Paraformaldehyde and sodium nitrite were bought from Lobachemie, India. Primary antibodies for TH (SC-25269), iNOS (SC-651), and glial fibrillary acidic protein (GFAP; SC-33673) were purchased from Santa Cruz, Biotechnology (Santa Cruz, CA, United States), and the primary antibodies for TNF-α (ab1793) and NF-κB (ab16502) were obtained from Abcam Life Science, Biogenuix Medsystems Pvt. Ltd. (New Delhi, India), and secondary fluorescent-tagged antibodies such as Cy2 conjugated and Cy3 conjugated for immunohistochemistry were bought from Merck Millipore and Chemicon, respectively.

### Experimental Animals

Swiss albino male mice, obtained from the animal research facility of the Institute of Medical Sciences, Banaras Hindu University, Varanasi, India, weighing 25 ± 5 g were used to conduct the experiment. Mice were kept in clean polypropylene cages, in an air-conditioned animal house with constant light–dark cycles for 12 h. Water and a standard mice pellet diet were made fully accessible. Only six mice were kept per cage, and they were acclimatized in the laboratory conditions for about 1 week before the start of the experiment. The experiment was done by following the guidelines suggested by the Animal Ethics Committee of Banaras Hindu University, Varanasi, India.

### Experimental Design

After the mice adapted to the laboratory conditions, they were further divided into five groups containing six animals each. The first was used as a control group. In the second group, two doses of MPTP (30 mg/kg body weight) were injected (i.p.) and the second dose of MPTP was administered after 16 h of the first dose. The third, fourth, and fifth groups were first intoxicated with two doses of MPTP (30 mg/kg body weight) similar to the second group. After 1 week of the second dose of MPTP, they were orally treated with CGA, dissolved in sterile water (25, 50, and 100 mg/kg body weight of CGA respectively) once daily for 24 days.

### Behavioral Studies

After the dosing was completed, behavioral parameters were checked after seven days from MPTP intoxication and also used to study motor deficits in mice. Different tests such as rotarod, hanging, and narrow beam walking tests were used for the motor study.

### Rotarod Test

In the rotarod experiments, animals were trained for three consecutive days before starting the experiment at a fixed speed (5 rpm). The time it took for the mice to fall down was recorded, up to a maximum of 5 min. For each animal, the experiment was repeated four times, and the average time was calculated (Manna et al., 2006). The experiment was repeated after the completion of the treatment, and the time it took for the mice to fall was also recorded.

### Narrow Beam Walking Test

This test was done to assess the motor coordination of mice, which is necessary to check the balance while moving on the narrow beam. At first, animals were trained to walk on a stationary narrow wooden beam placed 100 cm above the floor (L100 cm × W1 cm). Time taken by the mice to cross over the beam was recorded, and the experiment was repeated thrice (Pisa, 1998).

### Hanging Test

On a horizontal grid, mice were placed and allowed to hold it firmly. The grid was then inverted so that the mice hanged upside down until they lose their grip and fall. Hanging time was recorded, and the experiment was repeated thrice (Mohanasundari et al., 2006)

### Biochemical Parameters

After checking different behavioral parameters, mice were sacrificed by cervical dislocation followed by decapitation with minimum pain. The brains from mice were taken out and stored immediately in ice for further use. For biochemical tests, dissection of the mice brain from different groups was done in ice cold conditions, and midbrain and striatal tissue were isolated and kept in -20°C until the biochemical tests were fully performed (Kumar et al., 2010) and were further homogenized in KCl buffer (Tris–HCl 10 mM, NaCl 140 mM, KCl 300 mM, ethylenediaminetetraacetic acid 1 mM, Triton-X 100 0.5%) at pH 8.0 supplemented with protease and phosphatase inhibitors. The tissue homogenates thus obtained were subjected to centrifugation at 12,000 × *g* for 20 min at 4°C to obtain supernatant. Activities of different antioxidant enzymes such as superoxide dismutase (SOD) and catalase and levels of lipid peroxidation and nitrite were assayed from the supernatant.

### Estimation of Lipid Peroxidation

Lipid peroxidation was measured according to the method used by Ohkawa et al. (1979) with few modifications. First, 10% tissue homogenate was mixed with 10% SDS and then 20% acetic acid was added. After that, 0.8% TBA was added, and then the reaction mixture was kept in a boiling water bath for 1 h. Further, the absorbance was recorded against control at 532 nm after the assay mixture was cooled and centrifuged by taking out the supernatant. Peroxidation of lipids was assessed in micromoles of malondialdehyde (MDA) per milligram protein.

### Estimation of Nitrite Levels

The nitrite content was measured according to the standard procedures (Granger et al., 1996). 10% tissue homogenate was taken and mixed with ammonium chloride and Griess reagent. The solution was allowed to stand for half an hour at 37°C, and then the absorbance was taken at 540 nm. The reaction mixture was incubated at 37°C for 30 min, and the absorbance of the supernatant was recorded at 540 nm. The nitrite content was calculated using a standard curve for sodium nitrite (10–100 μM) in terms of micromoles per milliliter.

### Estimation of Activity of Antioxidant Enzymes

The catalase activity was evaluated by measuring the rate of decomposition of its substrate hydrogen peroxide using spectrophotometer (Kumar et al., 2010). For this, the striatal and midbrain tissue homogenates were mixed with potassium dichromate and acetic acid (1:3) in a boiling water bath for 10 min, and OD was taken at 570 nm. Measurement of the enzyme activity was done in *n* moles/min/mg protein. The activity of SOD was assayed using NADH as a substrate (McCord and Fridovich, 1969). The absorbance of both tubes was read at 560 nm against reagent blank. Difference between reference and experimental OD of the sample gives the inhibition of Nitro blue tetrazolium chloride (NBT) reduction by an enzyme source. Protein was also estimated by the enzyme source. The unit of SOD enzyme activity was defined as the amount of enzyme required to inhibit the optical density at 560 nm of NBT reduction by 50% in 1 min under the assay conditions. SOD activity was expressed as unit per milligram of protein.

### Immunohistochemical Staining

Mice from each group were first anesthetized with diethyl ether and perfused intracardially with 0.9% saline (chilled) and 4% paraformaldehyde (chilled) prepared in 0.1 M phosphate-buffered saline (PBS), pH 7.4. Brains were taken out by decapitation and kept in 10% paraformaldehyde overnight and further transferred to sucrose solution. Immunohistochemical staining of TH, GFAP, TNF-α, iNOS, and NF-κB was performed in both SN and ST using the standard procedure (Gorbatyuk et al., 2008). For this, 20 μm thick six to eight brain sections were cut coronally at both SN and ST levels using a cryomicrotome (Leica, Wetzlar, Germany). The sections were then washed with 0.01% M PBS (pH 7.4) at 10-min interval and then blocked with 10% NGS in PBS 0.3% Triton-X 100 and 1% BSA in phosphate buffered saline with Triton X-100 (PBST), that is, blocking reagent for about 1 h. The sections were further incubated with polyclonal anti-mice antibody against TH in 1:1000 dilution, monoclonal anti-mouse against GFAP in 1:1000 dilution, monoclonal TNF-α in 1:700 dilution, polyclonal anti-rabbit NF-κB p65 antibody in 1:1000 dilution, and anti-mouse for 16 h at 4°C. Further, washing was done with PBS and 1% BSA–PBS, respectively, two times each to remove unbound primary antibodies and then incubated with Cy2-conjugated secondary antibody (for anti-mice primary) and Cy3-conjugated secondary antibody (for anti-rabbit primary) prepared in 1% BSA–PBS for 1 h at room temperature. Then sections were washed thrice with 1% BSA–PBS at 3-min interval, and then DAPI was added (1 μg/ml). Finally, sections were washed thrice with PBS and then mounted on slides using polyvinyl alcohol mounting medium with DABCO anti-fading (Fluka analytical). The images were taken under Nikon fluorescent microscope (Thermo Fisher Scientific). Immunofluorescence was analyzed by Image J software (NIH, United States). The results were reported as mean integrated fluorescent value in SN and ST.

### Real-Time Polymerase Chain Reaction (PCR) Analysis

Total RNA was isolated from the frozen tissues using the Trizol RNA isolation reagent (Invitrogen, Carlsbad, CA, United States) according to the manufacturer’s instructions. The RNA yield was quantified on Nanodrop 1000, and the RNA purity was determined based on the A260/A280 ratio. For cDNA preparation, 2 μg total RNA (kept equal for each amplification) was subjected to reverse transcription using 20U M-MLV reverse transcriptase (Fermentas, Germany), 1 X reverse transcriptase (RT) buffer, 20 mM dNTPs (New England Biolabs, United States), 20 U RNasin (Fermentas, Germany), 0.1 M dithiothreitol (DTT) with diethyl pyrocarbonate (DEPC)-treated water, and 100 ng of random hexamers (Fermentas, Germany). Gene expression profile analysis was done on ABI7500 Fast system. GAPDH was taken as endogenous control. PCR reaction was done according to the previously reported studies with few modifications. In brief, 10 μl of real-time mix contained 5 μl of SYBER green master mix (Applied Biosystem), 1 μl cDNA, 2 μl nuclease-free water, 0.5 μl each of forward and reverse primers, and 1 μl RNase inhibitor. PCR conditions were set with an initial incubation at 50°C for 2 min, followed by denaturation at 95°C for 10 min, and 40 cycles at 95°C for 15 s, 60°C for 1 min, and 72°C for 40 s (Tiwari et al., 2017). The abundance or declines of mRNA were normalized to the geometric average of endogenous control GAPDH for ΔCt. The fold change was calculated using 2-ΔΔCt method and reported as arbitrary unit.

### Statistical Analysis

The data were analyzed by one-way analysis of variance (ANOVA) using Student–Newman–Keuls test, and the fold changes of mRNA were analyzed by Student’s two-tailed *t*-test using Graph Pad Prism 7.0 software. The results are expressed as the means ± SEMs. *P*-values <0.05 were considered statistically significant.

## Results

### CGA Attenuated the MPTP-Induced Motor Impairments in Mice

**FIGURE 2:**
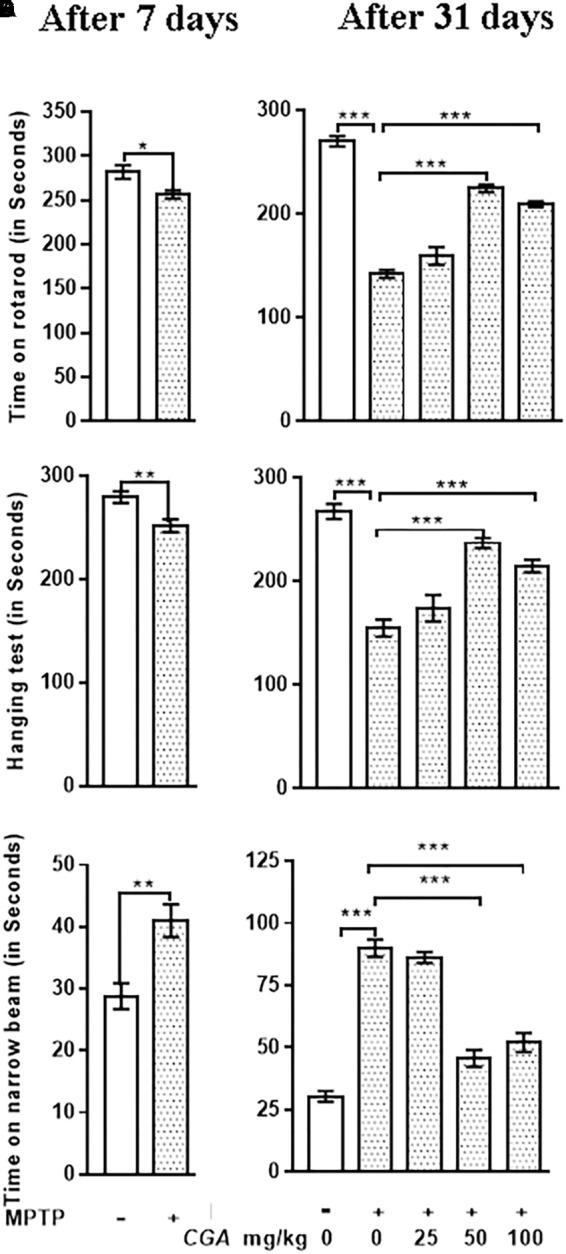
Effect of chlorogenic acid on behavioral parameters. **(A)** Rotarod test of mice was performed after 7 days from MPTP intoxication in control and MPTP groups. The time on the rotarod was found to be decreased in MPTP-intoxicated group compared with control. **(B)** After the completion of dosing, a significant improvement in the time of stay on rotarod was found in CGA-treated mice compared with the MPTP-intoxicated group. MPTP group shows reduced time of walking and staying on rotarod compared with the control group. **(C)** Hanging test was performed after 7 days from MPTP intoxication in control and MPTP groups. The hanging time was found to be reduced in MPTP-intoxicated groups compared with control. **(D)** After the completion of dosing, a significant improvement in the hanging time was observed in the CGA-treated group compared with the MPTP-treated group. MPTP group mice fall early compared with the control group. **(E)** Narrow beam walking test was performed after 7 days from MPTP intoxication in control and MPTP groups. The narrow beam walking time was found to be increased in MPTP-intoxicated groups compared with control. **(F)** In contrast, CGA treatment declines the walking time against MPTP-induced phenotype in mouse after the completion of dosing. Data are expressed in terms of mean ± SEM (*n* = 6), ^∗^*p* < 0.05, ^∗∗^*p* < 0.01, and ^∗∗∗^*p* < 0.001. CGA, chlorogenic acid; MPTP, 1-methyl-4-phenyl-1,2,3,6-tetrahydropyridine, SEM, standard error of mean.

Rotarod test explains about the balance and coordination of mice on a rotating beam (**Figures 2A,B**). After 7 days from MPTP intoxication, mice showed significant (*p* < 0.05) reduced time on the rotarod in comparison with the untreated control mice (**Figure 2A**). After day 31, time taken by the MPTP-intoxicated mice was considerably reduced (*p* < 0.001) compared with that of control (**Figure 2B**). After treatment with CGA (50 and 100 mg/kg body weight), the time taken by mice was significantly greater (*p* < 0.001) than that of MPTP-treated mice. Thus, 50 mg/kg body weight of CGA was optimum dose for treatment than the 25 and 100 mg/kg body weight of CGA. The degree of freedom and *F* values are *F*(4,45) = 99.11, and *p* < 0.001 with significant results found between the groups. The results were non-significant between MPTP and 25 mg/kg body weight of CGA.

Hanging test was performed to assess the motility of Parkinsonian mice. After 7 days from MPTP intoxication, mice showed significant (*p* < 0.01) reduced gripping and hanging time in comparison with the untreated control mice (**Figure 2C**). After day 31, gripping and hanging were significantly reduced (*p* < 0.001) in MPTP-treated mice compared with that of control (**Figure 2D**), whereas the hanging time was increased (*p* < 0.001) after the treatment of MPTP-intoxicated mice with CGA (50 and 100 mg/kg mg/kg body weight). The degree of freedom and *F* values are *F*(4,45) = 30.42, and *p* < 0.001 with significant results were found between the groups. The results were non-significant between MPTP and 25 mg/kg body weight of CGA.

The time taken by the mice to cross the narrow beam was significantly increased after 7 days from MPTP intoxication (*p* < 0.01) and after the completion of experiment (*p* < 0.001) in MPTP-intoxicated mice compared with that of control (**Figures 2E,F**). The walking time was significantly reduced (*p* < 0.001) on treatment with CGA (50 mg/kg body weight) in MPTP-induced mice. Accordingly, 50 mg/kg body weight of CGA (**Figures 2B,D,F**) was the optimum dose for treatment than the 25 and 100 mg/kg body weight of CGA. The degree of freedom and *F* values are *F*(4,45) = 70.63, and *p* < 0.001 with significant results were found between the groups. The results were non-significant between MPTP and 25 mg/kg body weight of CGA.

### Biochemical Analysis

Biochemical tests were performed after behavioral studies to see the effect of CGA on the level of antioxidants.

### CGA Inhibits Lipid Peroxidation and Nitrite Level in MPTP-Intoxicated Mice

**FIGURE 3:**
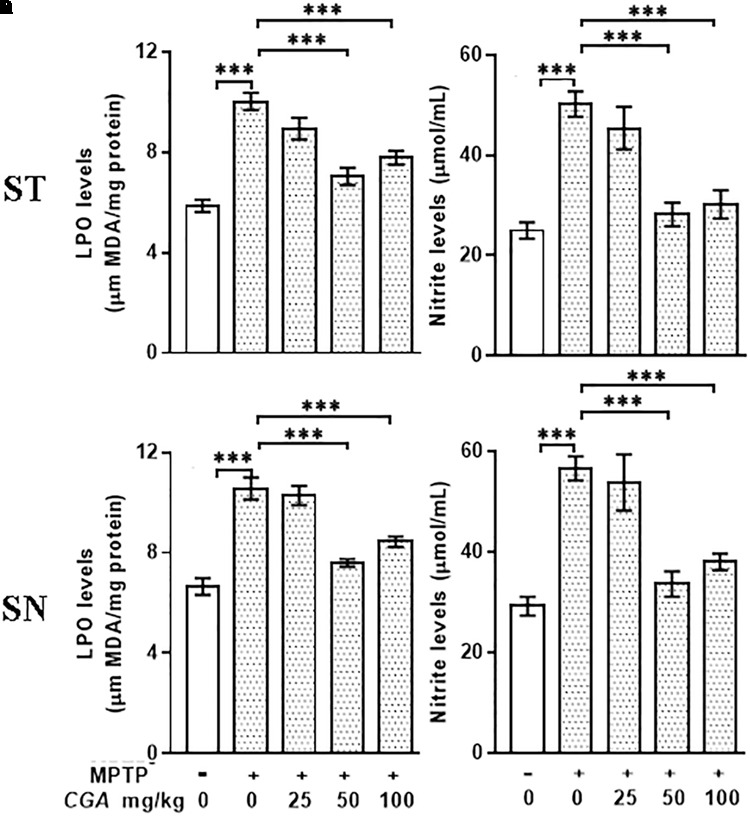
Estimation of MDA and nitrite levels: **(A–C)** MDA and **(B–D)** nitrite levels measured in striatal and substantia nigra regions of brain. MPTP injection caused significant increase in MDA and nitrite levels compared with the control group. CGA treatment alleviates MPTP-induced increased levels of MDA and nitrite compared with the MPTP-injected mice. Values are expressed as mean ± SEM (*n* = 6). ^∗^*p* < 0.05, ^∗∗^*p* < 0.01, and ^∗∗∗^*p* < 0.001. CGA, chlorogenic acid; MPTP, 1-methyl-4-phenyl-1,2,3,6-tetrahydropyridine; MDA, malondialdehyde; SEM, standard error of mean.

The level of MDA was measured to examine the effect of CGA on peroxidation of lipids in ST and SN (**Figures 3A,C**). The degree of freedom and *F* values of ST [*F*(4,20) = 23.43, *p* < 0.001] and SN [*F*(4,20) = 27.33, *p* < 0.001] with significant results were found between the groups. The results were non-significant between MPTP and 25 mg/kg body weight of CGA. Lipid peroxidation was considerably (*p* < 0.001) increased in MPTP-induced mice compared with that of control in both regions of brain. While on treatment with CGA, the MDA level was found to be decreased in the MPTP group (**Figures 3A,C**). MDA level was significantly reduced (*p* < 0.001) in 50 and 100 mg/kg body weight of CGA compared with the MPTP-intoxicated group.

The nitrite level was estimated within the ST and SN regions of different treatment groups. The degree of freedom and *F* values of ST [*F*(4,20) = 15.53, *p* < 0. 001] and SN [*F*(4,20) = 15.4, *p* < 0.001] with significant results were found between the groups. The results were non-significant between MPTP and 25 mg/kg body weight of CGA. In our study, the NO level was found to be significantly elevated (*p* < 0.001) within both the regions in Parkinsonian mice compared with that of control (**Figures 3B,D**). The NO level was significantly reduced (*p* < 0.001) in CGA (50 and 100 mg/kg body weight)-treated group compared with the MPTP-induced mice group. When MPTP-intoxicated groups were treated with three different doses of CGA, 50 mg/kg body weight of CGA has shown more efficacy rather than that of 25 and 100 mg/kg body weight groups of CGA.

### CGA Modulates the Activity of the Antioxidant Enzymes

**FIGURE 4:**
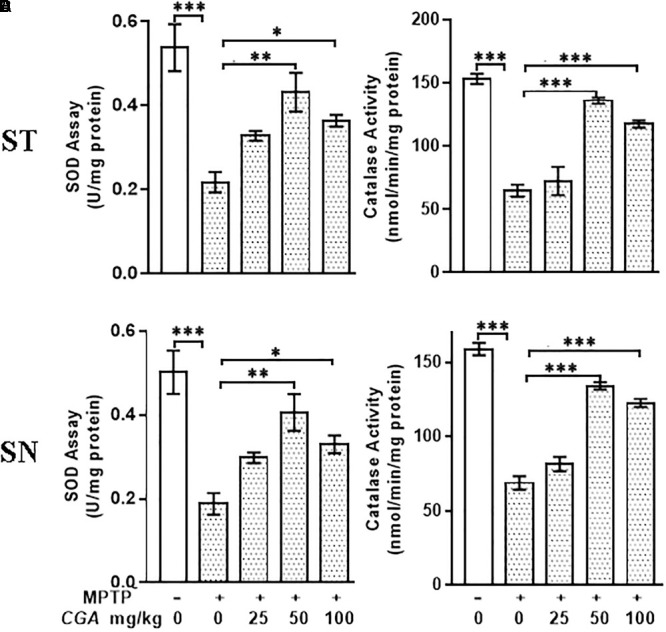
Estimation of activity of SOD and catalase. **(A–C)** Superoxide dismutase (SOD) and **(B–D**) catalase (CAT) activities measured in striatum and substantia nigra regions of the brain. MPTP injection caused significant increase in SOD and CAT activities in MPTP mice compared with the control group. CGA treatment in MPTP group increases SOD and CAT activities compared with the MPTP-injected animal group. Values are expressed as mean ± SEM (*n* = 6). ^∗^*p* < 0.05, ^∗∗^*p* < 0.01, and ^∗∗∗^*p* < 0.001. CGA, chlorogenic acid; MPTP, 1-methyl-4-phenyl-1,2,3,6-tetrahydropyridine; SOD, superoxide dismutase; CAT, catalase; SEM, standard error of mean.

In this study, the antioxidants such as SOD and catalase have effectively scavenged the ROS produced within the midbrain and ST. To study this, the activities of SOD and catalase were measured in ST and SN with the degree of freedom and *F* values of ST [SOD; *F*(4,20) = 11.46, *p* < 0.001] and SN [SOD; *F*(4,20) = 11.79, *p* < 0.0001] with significant results between the groups. The results were non-significant between MPTP and 25 mg/kg body weight of CGA. In the case of MPTP-intoxicated mice groups, SOD activity was found to be significantly decreased (*p* < 0.001) compared with that of control group (**Figures 4A,C**). However, on CGA treatment, the activity of SOD was seen to be significantly elevated mainly in the case of 50 mg/kg body weight of CGA treatment group (*p* < 0.01; **Figures 4A,C**).

Variation in the activity of catalase was found in different groups (**Figures 4B,D**) with the degree of freedom and *F* values of ST [*F*(4,20) = 92.31, *p* < 0.001] and SN [*F*(4,20) = 43.19, *p* < 0.001] with significant results. The results were found to be non-significant between MPTP and 25 mg/kg body weight of CGA. Compared with the control group, significant decrease (*p* < 0.001) in catalase activity was seen in ST and SN of MPTP-injected mice group. The treatment with CGA has, however, caused the elevation in the activity of catalase compared with the MPTP group, in which 50 and 100 mg/kg body weight of CGA have shown a significant increase in catalase activity (*p* < 0.001) compared with the MPTP-induced group (**Figures 4B,D**). Thus, the dosage of 50 mg/kg body weight of CGA was optimum dose for the treatment than that of 25 and 100 mg/kg body weight of CGA.

### Immunohistochemical Analysis

#### CGA Averts MPTP-Induced DA Neuronal Loss in SN and Decrease of Striatal DA Nerve Terminal Density

**FIGURE 5:**
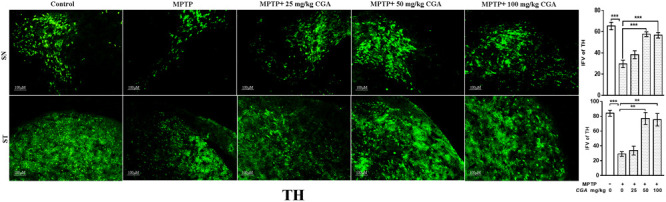
Immunohistochemistry of tyrosine hydroxylase in the SN and striatum of mice. With 10 × magnifications after staining. The expression of TH and nerve terminal density were significantly decreased in the SN and ST of MPTP-injected mice compared with the control group, whereas the expression of TH significantly increased in CGA-treated group compared with MPTP-injected mice. Values are expressed as mean ± SEM (*n* = 3). ^∗^*p* < 0.05, ^∗∗^*p* < 0.01, and ^∗∗∗^*p* < 0.001. CGA, chlorogenic acid; MPTP, 1-methyl-4-phenyl-1,2,3,6-tetrahydropyridine; SN, substantia nigra; TH, tyrosine hydroxylase; ST, striatum; SEM, standard error of mean; IFV, integrated fluorescent value.

Tyrosine hydroxylase immunostaining was done to study the expression of TH in DA neurons in the SN and ST regions of mice brain (**Figure 5**) with the degree of freedom and *F* values of SN [*F*(4,10) = 22.22, *p* < 0.001] and ST [*F*(4,10) = 17.37, *p* < 0.001] with significant results between the groups. The results were non-significant between MPTP and 25 mg/kg body weight of CGA. On MPTP intoxication, the expression of TH was reduced significantly (*p* < 0.001) compared with the control group in SN (**Figure 5**). Similarly, TH-positive nerve terminals were also reduced in the MPTP group more significantly (*p* < 0.001) compared with that of control (**Figure 5**). Treatment with CGA caused increase in TH immunostaining in SN and ST, respectively. More significant results were found in 50 and 100 mg/kg body weight of CGA showing increased TH-immunostaining in DA neurons and nerve terminals in SN (*p* < 0.001) and ST (*p* < 0.01), respectively.

#### CGA Inhibited the Activation of GFAP

**FIGURE 6:**
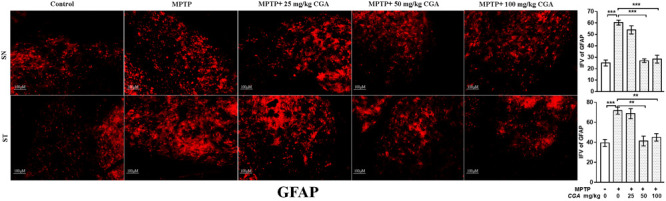
IHC of glial fibrillary acidic protein in the SN and striatum of mice. With 10 × magnifications after staining. The expression of GFAP-positive astrocytes was significantly increased in the SN and striatum of MPTP-injected mice compared with the control group, whereas CGA treatment decreased the expression of GFAP-positive astrocytes in MPTP+CGA group compared with the MPTP group. ^∗^*p* < 0.05, ^∗∗^*p* < 0.01, and ^∗∗∗^*p* < 0.001. CGA, chlorogenic acid; MPTP, 1-methyl-4-phenyl-1,2,3,6-tetrahydropyridine; SN, substantia nigra; ST, striatum; GFAP, glial fibrillary acidic protein; SEM, standard error of mean; IFV, integrated fluorescent value.

Increased oxidative stress and inflammation within astroglia are indicated by the enhanced expression of GFAP (**Figure 6**) with the degree of freedom and *F* values of SN [*F*(4,10) = 36.45, *p* < 0.001] and ST [*F*(4,10) = 13.86, *p* < 0.001] with significant results between the groups. The results were non-significant between MPTP and 25 mg/kg body weight of CGA. The expression of GFAP was considerably increased in the MPTP-intoxicated group compared with control in both SN and ST (**Figure 6**), indicating severe astrogliosis in the SN (*p* < 0.001) and ST (*p* < 0.001) as indicated by increased GFAP immunostaining. The treatment of different doses of CGA has reduced the expression of GFAP as shown by reduced GFAP immunostaining, of which 50 and 100 mg/kg body weight have shown more significant effects in SN (*p* < 0.001) and ST (*p* < 0.01) than that of 25 mg/kg body weight of CGA (**Figure 6**).

### Effect of CGA on the Expression of Inflammatory Mediators (TNF-α and iNOS)

**FIGURE 7:**
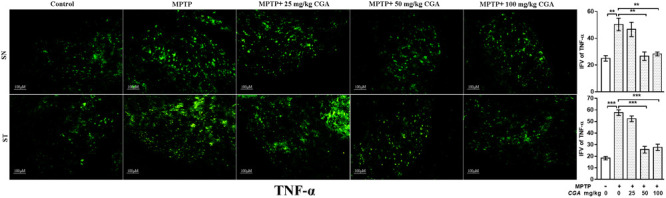
IHC of TNF-α in SN and striatum of mice. With 10 × magnifications after staining. The level of TNF-α was found to be increased in the SN and striatum of MPTP-treated mice compared with control mice, whereas CGA administration to MPTP-injected mice showed moderate staining of TNF-α in the MPTP+CGA mice compared with MPTP-injected mice. Values are expressed as mean ± SEM (*n* = 3). ^∗^*p* < 0.05, ^∗∗^*p* < 0.01, and ^∗∗∗^*p* < 0.001. CGA, chlorogenic acid; MPTP, 1-methyl-4-phenyl-1,2,3,6-tetrahydropyridine; SN, substantia nigra; ST, striatum; TNF-α, tumor necrosis factor; SEM, standard error of mean; IFV, integrated fluorescent value.

**FIGURE 8:**
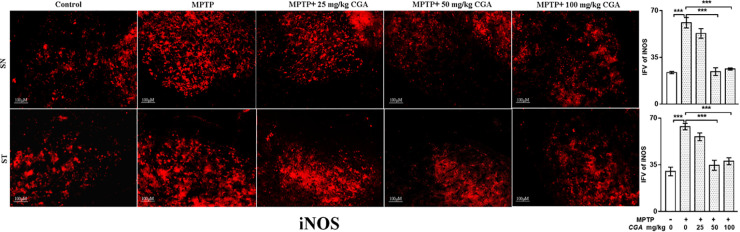
IHC of iNOS in the SN and striatum of mice. With 10 × magnifications after staining. The increased expression of iNOS in the SN and striatum was found in the MPTP-treated mice compared with control mice, whereas CGA supplementation reduced the expression of iNOS mice compared with MPTP-intoxicated mice. Values are expressed as mean ± SEM (*n* = 3). ^∗^*p* < 0.05, ^∗∗^*p* < 0.01, and ^∗∗∗^*p* < 0.001. CGA, chlorogenic acid; MPTP, 1-methyl-4-phenyl-1,2,3,6-tetrahydropyridine; SN, substantia nigra; ST, striatum; iNOS, inducible nitric oxide synthase; SEM, standard error of mean; IFV, integrated fluorescent value.

The effect of CGA on different inflammatory markers such as TNF-α and iNOS was studied in both SN and ST. TNF-α (*p* < 0.01 in SN and *p* < 0.001 in ST, **Figure 7**) and iNOS (*p* < 0.001 in SN and *p* < 0.001 in ST, **Figure 8**) expressions were found to be significantly increased in the MPTP-intoxicated group compared with that of control. 50 and 100 mg/kg body weight of CGA have significantly reduced the expression of TNF-α (*p* < 0.01 in SN and *p* < 0.001 in ST, **Figure 7**) and iNOS (*p* < 0.001 in SN and *p* < 0.001 in ST, **Figure 8**) compared with that of MPTP-injected group. The degree of freedom and *F* values of SN [*F*(4,10) = 10.88, *p* < 0.01] and ST [*F*(4,10) = 51.77, *p* < 0.001] for **Figures 7, 8** in SN [*F*(4,10) = 42.8, *p* < 0.001] and ST [*F*(4,10) = 23.53, *p* < 0.0001] with significant results between the groups. The results were found to be non-significant between MPTP and 25 mg/kg body weight of CGA.

### CGA Inhibited the Activation of NF-κB

**FIGURE 9:**
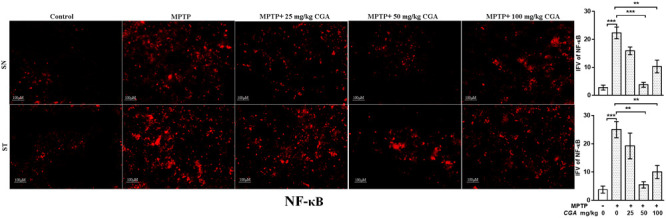
IHC of NF-κB in SN and striatum of mice. With 20 × magnifications after staining. NF-κB shows profound expression in the SN and striatum of MPTP-treated mice compared with the control group mice, while CGA treatment in MPTP mice shows moderate staining of NF-κB. Values are expressed as mean ± SEM (*n* = 3). ^∗^*p* < 0.05, ^∗∗^*p* < 0.01, and ^∗∗∗^*p* < 0.001. CGA, chlorogenic acid; MPTP, 1-methyl-4-phenyl-1,2,3,6-tetrahydropyridine; SN, substantia nigra; ST, striatum; NF-κB, Nuclear factor κB; SEM, standard error of mean; IFV, integrated fluorescent value.

Further, the expression of NF-κB was investigated in SN and ST regions of mice brain. As shown in **Figure 9**, the degree of freedom and *F* values of SN [*F*(4,10) = 26.24, *p* < 0.001] and ST [*F*(4,10) = 13.26, *p* < 0.001] with significant results were found between the groups. The results were found to be non-significant between MPTP and 25 mg/kg body weight of CGA. NF-κB immunostaining was found to be increased in the SN (*p* < 0.001, **Figure 9**) and ST (*p* < 0.001, **Figure 9**) in the MPTP-intoxicated group compared with that of control. CGA administration has inhibited the activation of NF-κB in the nigrostriatal region, of which 50 and 100 mg/kg body weight of CGA have markedly (*p* < 0.001 in SN and *p* < 0.01 in ST, **Figure 9**) reduced the activation of NF-κB in mice compared with MPTP-injected mice.

### Real-Time PCR Analysis: Effect of CGA on mRNA Expression of Inflammatory Cytokines

**FIGURE 10:**
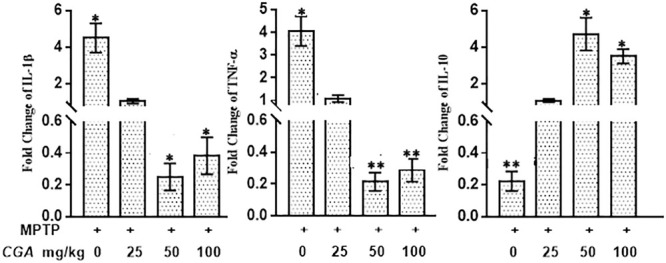
Effect of CGA on mRNA expression levels of inflammatory cytokines: IL-1β, TNF-α, and anti-inflammatory cytokine; IL-10 in PD mice model. In the absence of CGA, the MPTP-intoxicated mice brain tissue showed significantly enhanced expression levels of TNF-α and IL-1β with downregulated IL-10 levels compared with that of control. On supplementation with CGA at 50 and 100 mg/kg body weight concentration, the MPTP mice brain tissue showed significantly enhanced expression levels of IL-10 and decreased levels of TNF-α and IL-1β compared with the untreated MPTP-intoxicated mice. Values in the graph are represented as mean ± SEM with the level of significance ^∗^*p* < 0.05 and ^∗∗^*p* < 0.01. CGA, chlorogenic acid; MPTP, 1-methyl-4-phenyl-1,2,3,6-tetrahydropyridine; SN, TNF-α, tumor necrosis factor; SEM, standard error of mean.

The mRNA expression of the inflammatory cytokines was assessed by measuring the mRNA level of IL-1β, TNF-α, and IL-10 in different experimental groups. The anti-inflammatory effect of CGA on the mRNA expression of different cytokines such as IL-1β, TNF-α, and IL-10 in SN of MPTP-intoxicated mice has been shown in **Figure 10**. It can be clearly seen in **Figure 10** that mRNA levels of TNF-α (*p* < 0.05) and IL-1β (*p* < 0.05) were significantly increased, whereas the mRNA levels of anti-inflammatory cytokine IL-10 (*p* < 0.01) were seen to be downregulated in MPTP-intoxicated mice compared with that of control. Whereas CGA (50 mg/kg body weight) administration has potently reduced the mRNA expression of TNF-α (*p* < 0.05) and IL-1β (*p* < 0.01), while the mRNA expression of IL-10 was significantly increased (*p* < 0.05) compared with that of MPTP.

## Discussion

Our study has reported the antioxidative and anti-inflammatory activities of CGA by effectively scavenging ROS and RNS in MPTP-intoxicated mice. Oxidative stress and inflammation in neurons have been reported to play critical role in PD pathogenesis (Tansey et al., 2007). Microglial cells may become activated on inflammation and induce the process of neuronal degeneration through the production of ROS, thereby releasing pro-inflammatory cytokines, increasing NO levels and superoxides, and elevating iNOS expression rendering deleterious effects on DA neurons (Hirsch et al., 2003; Sanchez-Pernaute et al., 2004). Also, the usage of anti-inflammatory drugs to suppress neuroinflammation to alleviate DA neurodegeneration has been seen in different models of PD (Choi et al., 2005). Researchers have particularly focused to study the antioxidant properties of CGA as it crosses the blood–brain barrier whether in its pure form or as its metabolite (Ito et al., 2008; Chu et al., 2009; Kumar et al., 2018). Throughout our study, we tried to suggest the novel role of CGA by inhibiting the neuroinflammation by decreasing the production of ROS and release of pro-inflammatory cytokines and suppressing the activation of astrocytes by downregulating the expression of NF-κB.

We have used MPTP-induced mice as emphasized in many studies that MPTP acts as the best toxin-based animal model of PD representing all the pathological hallmarks (Langston et al., 1983). MPTP is converted to its toxic metabolite MPP^+^ (1-methyl-4-phenyl-pyridinium) by the enzyme monoamine oxidase B in astrocytes, further taken up by neurons through DA transporters. MPP^+^ causes its inhibitory action by entering into the mitochondria and blocking complex I function by which the first step in electron transport chain gets hindered. Hence, oxidative phosphorylation gets negatively affected by causing mitochondrial death due to oxidative stress (Dauer and Przedborski, 2003; Przedborski and Vila, 2003; Przedborski et al., 2004).

We tried to focus on the mechanism involved in the therapeutic action of CGA in MPTP-intoxicated mice. MPTP by and large not only promotes motor deficits, but it also causes DA neuronal degeneration in rodents (Blum et al., 2001). Moreover, it also causes similar behavioral, immunohistological, and biochemical effects to the human patients with PD. Further, MPTP treatment creates behavioral, neurochemical, and immunohistological characteristics very similar to patients with PD (RajaSankar et al., 2009). Among different doses of CGA, 50 mg/kg body weight dose is found to be more effective compared with 25 and 100 mg/kg body weight dose of CGA as shown by different behavioral, biochemical, immunohistochemical, and qPCR analyses. This study deals with the comparison between different doses of CGA in MPTP-intoxicated mice on the basis of TH immunoreactivity, neurobehavioral analysis, biochemical parameters, immunohistochemistry, and at the transcriptional level through qPCR analysis. In comparison to previous studies (Singh et al., 2009) showing motor impairment induced by MPTP, our study too showed similar results by conducting different behavioral parameters such as rotarod, hanging, and narrow beam walking tests. Our results have suggested that CGA treatment has remarkably reduced motor impairment by improving postural instability with more coordinated movements and effectively rescued motor deficits induced by MPTP.

Abnormal increase in the lipid peroxidation level has been seen in postmortem brains of patients with PD (Jenner and Olanow, 1996; Niranjan, 2014). MDA level is measured to check the level of lipid peroxidation, which acts as a marker of peroxidation of membrane phospholipids, due to oxidative stress (Maffei-Facino et al., 1995; Jose et al., 2001). In this study, lipid peroxidation was significantly increased due to MPTP intoxication. In contrast, upon treatment with CGA, the level was appreciably reduced indicating the scavenging property of CGA to remove ROS and reduce oxidative stress.

The increased oxidative stress within the cell is usually taken care of by the naturally occurring endogenous defense system present in it. The antioxidants here constitute enzymes such as CAT and SOD and non-enzymatic molecule GSH (Sutachan et al., 2012; Anderson and Maes, 2014; Celardo et al., 2014). The disturbance in the defense system has been recognized in PD brains (Anderson and Maes, 2014; Celardo et al., 2014). This defense system helps in scavenging superoxides and other intermediates of oxygen but up to normal threshold level. When the threshold level is crossed, this system fails to rescue the cell from oxidative stress. Enzyme SOD is usually responsible for converting superoxides into H_2_O_2_ (Zelko et al., 2002), which is further converted to water and oxygen through decomposition by CAT (Switala and Loewen, 2002). In our study, MPTP lesion has significantly reduced the activity of SOD and CAT. Upon CGA treatment, this effect was reversed, and the antioxidant defense mechanism was restored. The role of NO in contributing the hazardous effect of MPTP is reported by many studies. The NO level has been seen to be elevated upon MPTP intoxication, further enhancing DA neuronal damage (Jackson-Lewis and Smeyne, 2005). NO can block the initial respiration step by combining with superoxide anion (O^2-^), thereby producing highly toxic peroxynitrite oxidant (ONOO^-^). Our findings also reveal the involvement of NO in neurodegeneration, showing an elevated level of NO by MPTP intoxication, which was further reduced by CGA treatment.

The main finding of our study deals with the increment in TH immunoreactivity in DA neurons, conferred by CGA administration in MPTP-intoxicated mice. Measurement of TH-immunoreactivity is therefore performed to check the function of DA neurons and nerve fibers present in SN and ST, respectively (Lee J.M. et al., 2012). Reduction in TH immunoreactivity in SN has been observed upon MPTP challenge through immunohistochemical analysis. MPTP has led to significant downregulation of TH expression in SN and its projected nerve terminals in ST, which is supported by numerous reports (Lee J.M. et al., 2012). The decreased expression of TH in SN and its terminals in ST is considered as one of the pathological hallmarks in the case of PD. This condition was significantly reversed by administration of CGA to MPTP-intoxicated mice, which has significantly rescued the TH expression and preserved integrity of nerve terminals.

Inflammation plays a central role in the pathogenesis of PD. This fact is also supported by various studies done using rodent model of PD and postmortem PD brains that have shown the presence of neuro-inflammatory cascade involved in neurodegeneration (Mogi et al., 1994; McCoy et al., 2008; Hirsch and Hunot, 2009). Inflammation induced either by the glial cell activation and proliferation or by the production of cytokines is said to be one of the pathogenic components of brain damage. The pro-inflammatory and cytotoxic factors usually help cells by rescuing them from invading pathogen. However, under continued production, they might lead to neurotoxic effects resulting in the damage of neurons. Cytokines are primarily expressed within the glial cells of CNS (Teismann and Schulz, 2004). Not only DA neuronal loss is caused by reactive microglia but also reactive astrocytes show their detrimental effect to the neuron survival (Finsterwald et al., 2015). Astrocytes also respond to inflammatory stimulations such as IL-1β, LPS, and TNF-α by producing pro-inflammatory cytokines both *in vitro* and *in vivo*, like microglia, shown by various studies (Saijo et al., 2009; Tanaka et al., 2013). The increased expression of GFAP within astroglial cells, indicating astrogliosis marked by hypertrophy of cell body and extensions, has been reported in various models of PD. In accordance with the previous reports (Litteljohn et al., 2011), our study also shows that MPTP challenge leads to increased astrogliosis seen by elevated expression of GFAP, indicating increased production of ROS and neuroinflammation (Lee et al., 2009). In contrast, administration of CGA has led to attenuation of the increased level of GFAP, thereby blocking astrocyte activation. Thus, it clearly suggests that CGA potentially rescued the DA neurons from damage by ameliorating activation of astrocyte, an initial step of neurodegeneration.

To investigate the anti-inflammatory role of CGA, the levels of various inflammatory components were studied. Cytokines, such as TNF-α and IL-1β, regulate the inflammatory responses by playing important roles in host defence, infection, and pathogenesis of the disease (Boudjellab et al., 2000; Lee et al., 2006). Our study also indicates that MPTP treatment has increased the level of pro-inflammatory molecules such as TNF-α and iNOS in SN and ST, whereas administration of CGA to MPTP-intoxicated mice has significantly reduced the level of TNF-α and iNOS.

In the present study, the expression of NF-κB was found to be increased in MPTP-treated mice. On CGA treatment, decrement in the expression of NF-κB was observed, showing its ability to reverse inflammatory cascade. NF-κB is a ubiquitous transcription factor found in almost all animal cell types (Figuera-Losada et al., 2014). This transcription factor plays an important role in activating the genes responsible for expressing pro-inflammatory cytokines. It has been proved by several studies that translocation of NF-κB from cytoplasm to nucleus leads to the overexpression of inflammatory mediators such as IL-1β, IL-6, iNOS, and TNF-α (di Meglio et al., 2005; Wang et al., 2017). Reportedly, NF-κB activation was found prominently in patients with PD and in SN of MPTP-treated mice (Ghosh et al., 2007). Upregulation of NF-κB is usually linked with the destruction of neurons. On CGA treatment, decrement in the expression of NF-κB was observed, showing its ability to reverse inflammatory cascade after MPTP intoxication. Excessive inflammation that can cause severe damage to the cells is generally counteracted by the production of the anti-inflammatory cytokine IL-10 by many immune cells (Moore et al., 2001; Lobo-Silva et al., 2016). IL-10 helps in maintaining neuron viability and regulates inflammation (Strle et al., 2001). Moreover, IL-10 prevents the apoptosis by enhancing the expression of anti-apoptotic factors and attenuating the expression of pro-apoptotic factors, thus, playing an important role in CNS level. IL-10 inhibits the production of cytokines by microglia and thus inhibits excessive neuroinflammation (Balasingam and Yong, 1996; Ledeboer et al., 2002). In accordance with these studies, our study also shows the reduced production of anti-inflammatory cytokine IL-10 in MPTP-intoxicated mice, whereas it was significantly enhanced on CGA treatment.

In general, our study deals with the therapeutic effect of CGA in MPTP-intoxicated mice, which is solely caused by its antioxidative and anti-inflammatory activities. Our findings show that after CGA treatment, the increased expression of existing TH cells did not get degenerated due to MPTP intoxication. Based on these results, future studies are warranted to assess the TH-positive cell counts besides their exact molecular mechanism by which CGA restores the antioxidant capacity or inflammatory response in MPTP-intoxicated mice.

## Conclusion

To sum up, our findings suggest that CGA can be used as a potent anti-inflammatory agent in preventing the neurodegeneration in PD. It confers its effects mainly by downregulating the expressions of iNOS, TNF-α, and NF-κB in activated glial cells, thereby inhibiting neuroinflammation through its elevated anti-inflammatory and antioxidant activities. Thus, the anti-inflammatory activity along with the potent antioxidant properties shown by CGA can be used in treating the inflammatory condition in the case of PD.

## Author Contributions

SSS, SNR, HB, and WZ designed and performed the experiments and co-wrote the manuscript. NT and RS performed the RT-PCR experiments. MG, GK, and RP statistically analyzed the study. SPS conceived, designed, and supervised the complete study.

## Conflict of Interest Statement

The authors declare that the research was conducted in the absence of any commercial or financial relationships that could be construed as a potential conflict of interest.
