# Corrigendum: Effect of chlorogenic acid supplementation in MPTP-intoxicated mouse

**DOI:** 10.3389/fphar.2023.1145172

**Published:** 2023-02-02

**Authors:** Saumitra S. Singh, Sachchida N. Rai, Hareram Birla, Walia Zahra, Gaurav Kumar, Mallikarjuna R. Gedda, Neeraj Tiwari, Ranjana Patnaik, Rakesh K. Singh, Surya P. Singh

**Affiliations:** ^1^ Department of Biochemistry, Institute of Science, Banaras Hindu University, Varanasi, India; ^2^ School of Biomedical Engineering, Indian Institute of Technology (BHU), Banaras Hindu University, Varanasi, India

**Keywords:** chlorogenic acid, Parkinson’s disease, oxidative stress, neuroinflammation, dopaminergic neuron, substania nigra

In the published article, there was an error in Figure 8 as published. The figure panels in **Figure 8** were erroneously duplicated. The corrected Figure 8 and its caption appear below.

**FIGURE 8:**
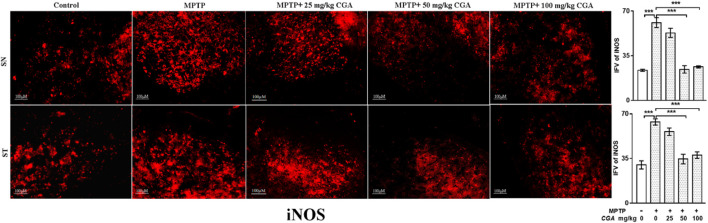
IHC of iNOS in the SN and striatum of mice. With 10× magnifications after staining. The increased expression of iNOS in the SN and striatum was found in the MPTP-treated mice compared to control mice, whereas CGA supplementation reduced the expression of iNOS in mice compared with MPTP-intoxicated mice. Values are expressed as mean SEM (n = 3). *p* < 0.05, ;*p* < 0.01, and *p* < 0.001. CGA, chlorogenic acid; MPTP, 1-methyl-4-phenyl-1,2,3,6-tetrahydropyridine; SN, substantia nigra; ST, striatum; iNOS, inducible nitric oxide synthase; SEM, standard error of mean; IFV, integrated fluorescent value.

The authors apologize for this error and state that this does not change the scientific conclusions of the article in any way. The original article has been updated.

